# A Family of Helminth Molecules that Modulate Innate Cell Responses via Molecular Mimicry of Host Antimicrobial Peptides

**DOI:** 10.1371/journal.ppat.1002042

**Published:** 2011-05-12

**Authors:** Mark W. Robinson, Sheila Donnelly, Andrew T. Hutchinson, Joyce To, Nicole L. Taylor, Raymond S. Norton, Matthew A. Perugini, John P. Dalton

**Affiliations:** 1 Infection, Immunity and Innovation (i3) Institute, University of Technology Sydney (UTS), Ultimo, Sydney, Australia; 2 Department of Biochemistry and Molecular Biology, Bio21 Molecular Science and Biotechnology Institute, University of Melbourne, Australia; 3 Monash Institute of Pharmaceutical Research, Monash University, Parkville, Australia; 4 Institute of Parasitology, McDonald Campus, McGill University, St. Anne de Bellevue, Quebec, Canada; National Institute of Allergy and Infectious Diseases and National Institutes of Health, United States of America

## Abstract

Over the last decade a significant number of studies have highlighted the central role of host antimicrobial (or defence) peptides in modulating the response of innate immune cells to pathogen-associated ligands. In humans, the most widely studied antimicrobial peptide is LL-37, a 37-residue peptide containing an amphipathic helix that is released via proteolytic cleavage of the precursor protein CAP18. Owing to its ability to protect against lethal endotoxaemia and clinically-relevant bacterial infections, LL-37 and its derivatives are seen as attractive candidates for anti-sepsis therapies. We have identified a novel family of molecules secreted by parasitic helminths (helminth defence molecules; HDMs) that exhibit similar biochemical and functional characteristics to human defence peptides, particularly CAP18. The HDM secreted by *Fasciola hepatica* (FhHDM-1) adopts a predominantly α-helical structure in solution. Processing of FhHDM-1 by *F. hepatica* cathepsin L1 releases a 34-residue C-terminal fragment containing a conserved amphipathic helix. This is analogous to the proteolytic processing of CAP18 to release LL-37, which modulates innate cell activation by classical toll-like receptor (TLR) ligands such as lipopolysaccharide (LPS). We show that full-length recombinant FhHDM-1 and a peptide analogue of the amphipathic C-terminus bind directly to LPS in a concentration-dependent manner, reducing its interaction with both LPS-binding protein (LBP) and the surface of macrophages. Furthermore, FhHDM-1 and the amphipathic C-terminal peptide protect mice against LPS-induced inflammation by significantly reducing the release of inflammatory mediators from macrophages. We propose that HDMs, by mimicking the function of host defence peptides, represent a novel family of innate cell modulators with therapeutic potential in anti-sepsis treatments and prevention of inflammation.

## Author Summary

The study of pharmacologically active peptides is central to the understanding of disease and the development of novel therapies. Of particular interest is the array of short cationic peptides, collectively known as antimicrobial (or host defence) peptides. In humans, the most widely studied host defence peptide is LL-37. This α-helical peptide is expressed by neutrophils, macrophages and mucosal epithelial cells and modulates the response of innate immune cells to pathogen-associated ligands (such as bacterial lipopolysaccharide). We have identified a novel family of molecules secreted by medically-important helminth pathogens (termed helminth defence molecules; HDMs) that exhibit striking structural, biochemical and functional similarities to host defence peptides, including LL-37. HDMs bind LPS preventing the activation of macrophages and the release of pro-inflammatory mediators that can cause sepsis. We propose that HDMs modulate the immune response via molecular mimicry of host defence peptides, thus creating a unique opportunity for novel anti-infective therapy.

## Introduction

The detection of microbial invasion by cells of the innate immune response (macrophages and dendritic cells; DCs) occurs via receptor recognition of specific microbial molecular patterns (pathogen-associated molecular patterns; PAMPS), such as the Gram-negative lipopolysaccharide (LPS) endotoxin. Interaction between cell receptors and bacterial PAMPS drives the early immune response and leads to maturation of anti-microbial responses manifested in the production of potent pro-inflammatory cytokines such as IL-6, IL-12 and TNF [1]. While the production of these cytokines, together with up-regulation of co-stimulatory molecules on DCs, macrophages, granulocytes and mast cells, is a critical step in the development of appropriate protective adaptive immune responses, an excessive inflammatory response can lead to sepsis, septic shock and death [2], [3].

Human defence peptides are potent signalling molecules released by cells of the innate immune system in response to cellular stimulation by microbes and pro-inflammatory mediators [4]. Multiple peptides are simultaneously secreted at the site of infection and/or inflammation and work cooperatively to perform broad-spectrum antimicrobial activities [5], [6]. While it is well recognized that defence peptides participate as direct antimicrobial effectors of innate immunity by disrupting bacterial membranes [7], more recent studies suggest that they are also important in the regulation of innate immune responses and are critical in protecting against the detrimental effects of an excessive innate inflammatory response. Thus, defence peptides have been shown to suppress LPS-mediated responses [3], [8], promote phagocytosis while inhibiting oxidant responses of neutrophils or monocytes [9], [10], and inhibit pro-inflammatory cytokine secretion by macrophages in the presence of bacteria or other non-specific inflammatory stimuli [6], [10].

It has been demonstrated experimentally that intestinal injury and systemic endotoxemia are two factors leading to morbidity in helminth infection of mice [11], [12]. Disruption to the barrier function of the intestinal epithelium and consequently the translocation of luminal antigens (bacteria and their toxins) into the circulation is common to many helminth parasites. Thus, infection with enteric nematodes is characterised by enhanced leakiness of the intestinal epithelium, mediated by activated mast-cells [13], which aids the expulsion of the parasite from the host but can lead to the movement of bacterial LPS into the portal circulation [14]. Even in non-enteric helminths, such as the schistosomes that reside in the mesenteric veins, damage caused by worm eggs traversing the intestinal epithelium can result in the systemic translocation of bacteria [11]; for example, *E. coli* are frequently detected in the mesenteric lymph nodes of *Schistosoma mansoni*-infected patients [15]. Biliary obstruction due to the growth of *Fasciola hepatica* worms in the bile ducts increases intraductal pressure leading to the disruption of hepatocellular tight junctions and subsequent translocation of *E. coli* and enterococcus [16], [17]. Despite such bacterial colonisation, potent host responses such as septicaemia are not common events during helminth infections. While the mechanism of resistance to septicaemia during helminth infection is poorly understood, it is known that innate immune cells rather than those of the adaptive immune system play an essential protective role. For example, mice deficient in the IL-4α receptor specifically on macrophages and neutrophils, but not T-cells, experienced high mortality (100%) associated with increased sepsis following infection with *S. mansoni* [11], [12].

Here we describe a novel family of proteins that is secreted by medically-important trematode pathogens. As these proteins display similar functional and biochemical characteristics to the human defence peptides, defensins and cathelicidins, we have termed them helminth defence molecules (HDMs). We have characterised the HDM secreted by *F. hepatica* (FhHDM-1), and show that proteolytic processing of this molecule by a co-secreted cathepsin L1 releases a 34-residue C-terminal fragment containing an amphipathic helix. This amphipathic peptide is conserved amongst the HDMs of the major trematode pathogens of humans and exhibits similar structural features to LL-37, an anti-microbial peptide proteolytically released from the human cathelicidin precursor, CAP18. Recombinant FhHDM-1 and a synthetic peptide corresponding to the amphipathic C-terminal fragment bind directly to LPS in a concentration-dependent manner, block its interaction with LPS-binding protein (LPB), and prevent it binding to the surface of macrophages. Furthermore, both FhHDM-1 and the amphipathic C-terminal peptide protect mice against LPS-induced inflammation by significantly reducing the release of inflammatory mediators from macrophages. We propose that HDMs represent a novel family of helminth modulators of host innate immunity that are responsible for preventing the induction of sepsis during infection.

## Results

### A novel family of helminth defence molecules (HDMs)

**Figure 1:**
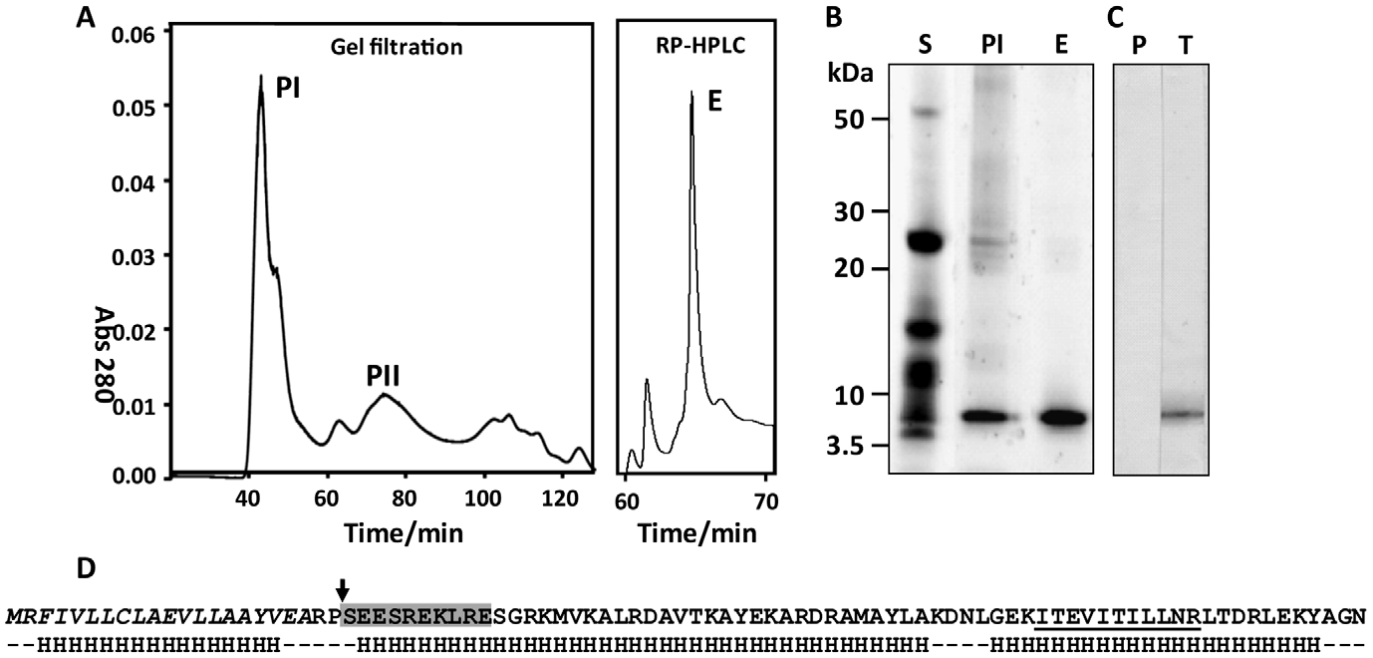
Identification and characterisation of native FhHDM-1. (A) Secretory proteins collected from adult *F. hepatica* following *in vitro* culture were separated by gel filtration and the resulting high molecular mass (>200 kDa) peak (peak I; PI) was separated further using reverse phase HLPC (RP-HPLC). Fractions collected following gel filtration and RP-HPLC were run on reducing 4–12% Bis-Tris gels (B) and showed that a prominent ∼ 6 kDa protein present in total adult secretory proteins (S) was enriched in PI and purified to homogeneity (>95%) following RP-HPLC (E). (C) Western blot of adult fluke secretions probed with an anti-FhHDM-1 antibody. P, pre-immune sera; T, test bleed. (D) N-terminal sequencing and LC-MS/MS analysis of the native ∼ 6 kDa protein generated peptide sequence information that allowed cloning of the cDNA, termed FhHDM-1. The primary amino acid sequence of FhHDM-1 derived from conceptual translation of the cDNA is shown. The predicted N-terminal signal peptide is shown in italics and the actual N-terminal of the native protein is shown by an arrow. The SEESREKLRE sequence generated by N-terminal sequencing is boxed in grey and a peptide (*m/z* 642.93; ITEVITILLNR) matched by LC-MS/MS following tryptic digest of the native protein is underlined. Secondary structure predictions using using PSIPRED [26], shown below the primary sequence, suggest the molecule is predominantly α-helical.

Fractionation by gel filtration chromatography of proteins secreted by adult *F. hepatica* yielded two major peaks, termed PI (>200 kDa) and PII (20–60 kDa) (Figure 1A). Because of different separation techniques employed in the present study the elution and protein profiles of PI and PII appear slightly different from those presented in our earlier studies [18]. We have shown previously that PI contains the anti-oxidant peroxiredoxin (Prx; [18], [19]). However, SDS-PAGE analysis revealed that this fraction contains another major component with a molecular mass around 8 kDa (Figure 1B). Whilst it is not fully understood why this 8 kDa protein is the most abundant species in the >200 kDa PI fraction, we suspect that it forms aggregates with other secretory proteins, including peroxiredoxin [18]. These aggregates are dispersed by RP-HPLC using acetonitrile in the mobile phase allowing the 8kDa protein to be isolated to homogeneity (Figure 1B, lane E). N-terminal sequencing of this band generated a single clean sequence (SEESREKLRE) that matched a putative peptide encoded by several *F. hepatica* ESTs (e.g. HAN3008-1e06.p1k) following tBLASTn analysis. This N-terminal sequence is identical to the first ten amino acids of a sequence reported by McGonigle *et al*. [20] within a high molecular mass fraction of *F. hepatica* secretory proteins, although the identity of the protein remained elusive until the present study.

**Figure 2:**
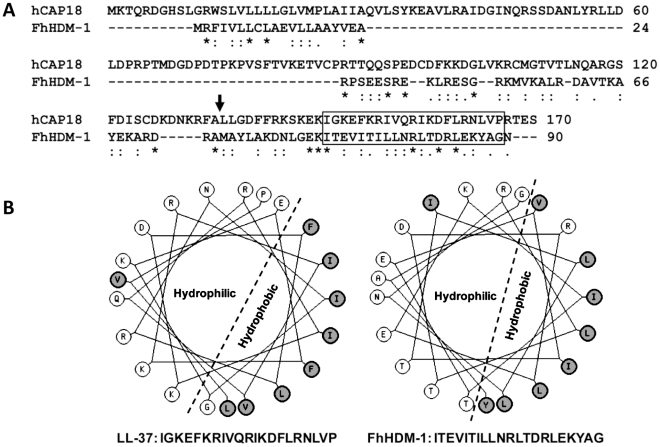
FhHDM-1 is structurally homologous with LL-37. (A) Primary sequence alignment of FhHDM-1 with the human LL-37 precursor, hCAP18. The LL-37 processing site is arrowed. (B) Helical wheel analysis shows that the conserved C-terminal hydrophobic regions boxed in (A) form amphipathic helices in both molecules.

Analysis of the conceptually translated *Fasciola* ESTs using SignalP [21] predicted a signal peptide of residues 1-20. However, N-terminal sequencing of the native protein showed that residues 1–22 were removed upon secretion (Figure 1D). RT-PCR was performed to verify the coding region of the cDNA; the resulting 222 bp cDNA included a 210 bp open reading frame encoding a 70 amino acid mature protein (Figure 1D; GenBank accession no. HQ456365).The mature protein was predicted from primary sequence to have a molecular mass of 8 kDa, which is close to that observed by SDS-PAGE (Figure 1B) and Western blot analysis of the native *F. hepatica* secretions using an anti-FhHDM-1 antibody (Figure 1C). Analysis of the FhHDM-1 primary sequence using InterProScan [22] did not detect any known functional domains or motifs. However, our analysis of the predicted secondary structure suggested structural homology with host defence peptides and in particular human CAP18/LL-37 (Figure 2) and, therefore, we termed the novel protein *F. hepatica* helminth defence molecule-1 (FhHDM-1).

**Figure 3:**
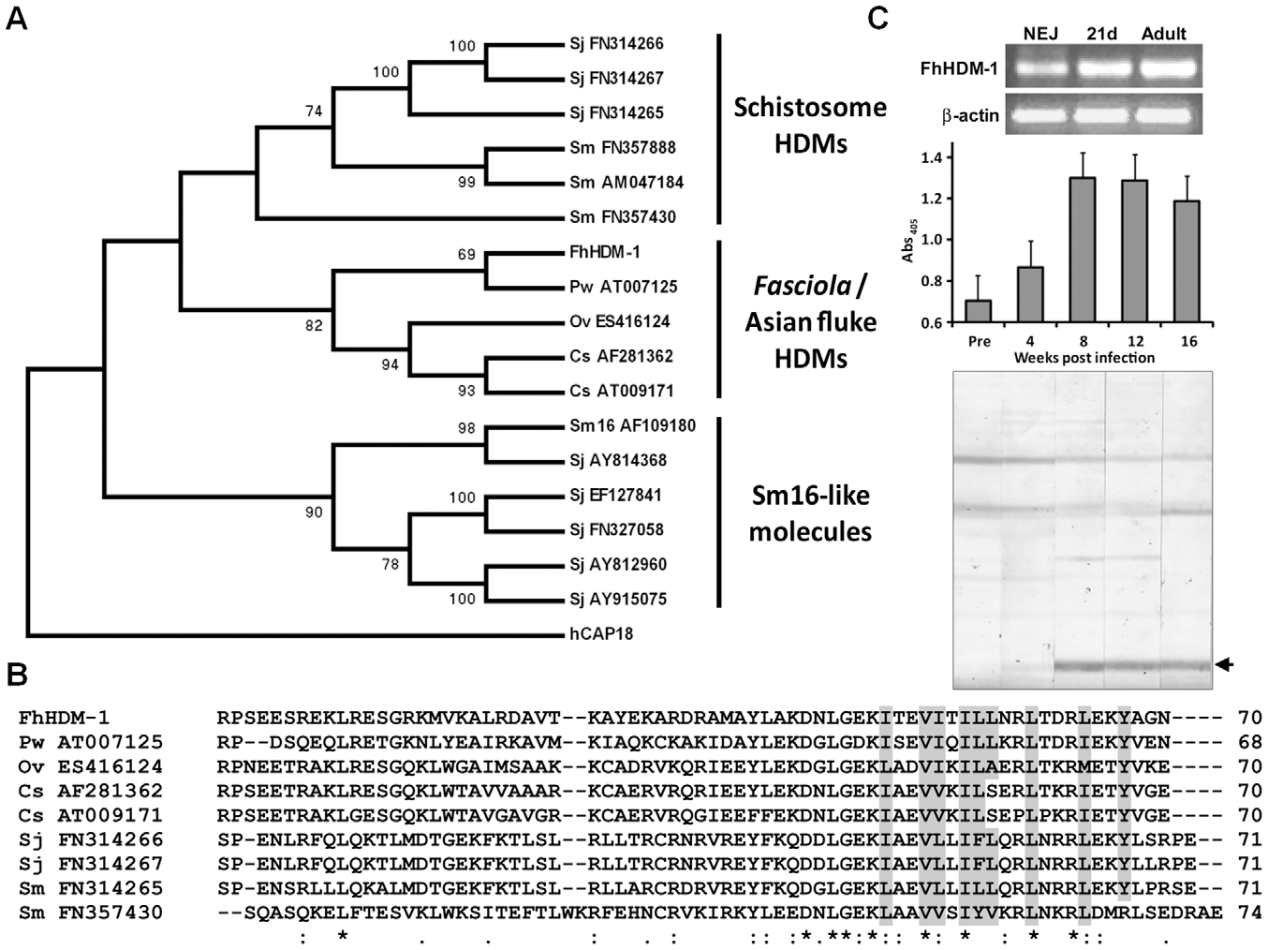
Phylogenetic relationships of the HDMs. (A) A bootstrapped (1000 trials) neighbour-joining phylogenetic tree showing the evolutionary relationship of HDM cDNA sequences from medically-important trematode pathogens. Numbers represent bootstrap values (given as percentages) for a particular node, and values greater than 65% are shown. The tree is rooted to human CAP18 (accession number NM_004345). Three major clades are shown corresponding to the Sm16-like molecules, the schistosome HDMs and HDMs from *Fasciola* and the Asian flukes. (B) Primary sequence alignment of selected members of the HDM clades. Conserved residues that contribute to the hydrophobic face of the amphipathic helix are shaded in grey. (C) Top panel. RT-PCR analysis of *FhHDM-1* expression in *F. hepatica* newly excysted juveniles (NEJ), 21-day immature flukes (21d) and adult worms (Adult). Amplification of constitutively expressed *F. hepatica* β-actin was performed as a positive control. Samples were separated by agarose gel electrophoresis and stained with ethidium bromide. Bottom panel. Immunogenicity of FhHDM-1 in *F. hepatica*-infected sheep. Pre-infection sera (Pre) and samples taken 4, 8, 12 and 16 weeks post-infection were analysed by ELISA and Western blot using an anti-FhHDM-1 antibody. Specific antibody responses were detected at week 4 with immunoblot staining stronger at weeks 8 and 12 after infection.

Analysis of cDNA and available genomic databases using tBLASTn [23] identified putative homologues of FhHDM-1 in related trematodes that are major pathogens of humans, including the liver flukes *Clonorchis sinensis* and *Opisthorchis viverrini*, the lung fluke *Paragonimus westermani*, and the blood flukes *S. mansoni* and *S. japonicum* (Figure 3A and B). The FhHDM-1 protein aligns with a 7 kDa protein (55% sequence identity) derived from a *C. sinsensis* cDNA [24] and with the C-terminal region (∼30% sequence identity) of the *S. mansoni* secreted protein Sm16 [25]. The evolutionary relationships of FhHDM-1 and the related trematode cDNA/EST sequences (17 in all) deposited in the public databases and at http://www.sanger.ac.uk/Projects/Helminths/was investigated at the molecular level by constructing a bootstrapped neighbour-joining tree (Figure 3A). A phylogenetic analysis revealed that these sequences separated into three clades which segregated the Sm16-like molecules from the HDMs which themselves were split into two clades corresponding to schistosome HDMs and those of *Fasciola* and the Asian flukes. Despite this segregation, all HDM clade members share a number of similarities including (a) a predicted N-terminal signal peptide (b) a predicted α-helical secondary structure, and (c) a largely hydrophobic C-terminal portion of approximately 35 residues that is the most conserved region of the molecule both within and between clades (Figure 3B).

The expression of the FhHDM-1 gene in NEJs, 21-day immature liver stage flukes and adult *F. hepatica* was determined by RT-PCR using the expression of the housekeeping gene β-actin as a loading control. FhHDM-1 was constitutively expressed in all three life-cycle stages (Figure 3C, top). The protein is also clearly secreted during all stages of host infection as an antibody response against FhHDM-1 was detected in the serum of experimentally-infected sheep corresponding to the migratory stages (4–8 weeks post-infection) and against mature worms residing in the bile ducts (>12 weeks post-infection) (Figure 3C, bottom).

### Native and recombinant FhHDM-1 possess significant α-helical structure

**Figure 4:**
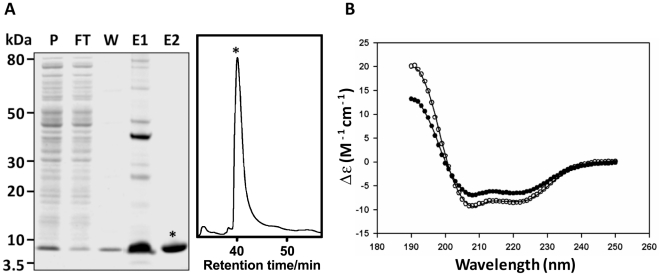
Expression and CD spectroscopy of recombinant FhHDM-1. (A) The full-length FhHDM-1 cDNA, minus the N-terminal signal peptide, was expressed in *E. coli* and the His-tagged recombinant was purified from cell lysates using Ni-NTA agarose (Qiagen). P, pre-column; FT, flow-through; W, wash, E1, imidazole eluate. Co-eluting proteins were removed by RP-HLPC resulting in recombinant FhHDM-1 of very high purity (E2). (B) CD spectra of recombinant 0.1 mg/mL^−1^ FhHDM-1 at pH 7.3. The wavelength scan was performed between 190 and 250 nm. The final spectrum (closed circles in the absence of 30% (v/v) TFE and open circles in the presence of 30% (v/v) TFE) is the average result from three scans measured at 20°C. The CONTINLL algorithm from the CDPro software package [27] produced the best fit (solid lines) against the SP29 protein database [28] with r.m.s.d. values for all samples ≤0.325. FhHDM-1 adopts a near identical solution structure in both native and recombinant form at both pH 4.5 and pH 7.3 (data not shown). The resulting secondary structure proportions are reported in Table S1.

Secondary structure predictions using PSIPRED [26] indicate that FhHDM-1 has a high propensity to form α-helices (Figure 1D). In order to demonstrate this experimentally, CD spectroscopy was used to estimate the secondary structure of both native (purified as described above) and recombinant FhHDM-1. Full-length FhHDM-1, lacking the predicted N-terminal signal peptide, was expressed in *E. coli* as a 9.2 kDa recombinant protein fused to a C-terminal His_6_-tag for purification. The recombinant FhHDM-1 protein was isolated to homogeneity from *E. coli* cell lysates by a combination of Ni-chelate affinity chromatography and RP-HPLC (Figure 4A).

CD spectroscopy was performed in solution in the presence or absence of 30% (v/v) TFE at pH 4.5 and pH 7.3. The resulting spectra display double minima at 208 nm and 222 nm, indicative of α-helical structure. In order to confirm this assertion, the CD spectra were fitted by nonlinear least squares regression using the CDPro software package employing the CDSSTR, CONTINLL and SELCON3 algorithms [27]. The best fit for each sample, resulting from the CONTINLL algorithm against the SP29 database [28], predicted both native and recombinant FhHDM-1 to have predominantly α-helical secondary structure in the presence and absence of 30% (v/v) TFE at both pH 4.5 and pH 7.3 (Table S1). Given their similarity, a CD spectrum representative for all samples analysed (recombinant FhHDM-1 at pH 7.3) is shown in Figure 4B.

### FhHDM-1 oligomerisation is enhanced at low pH

**Figure 5:**
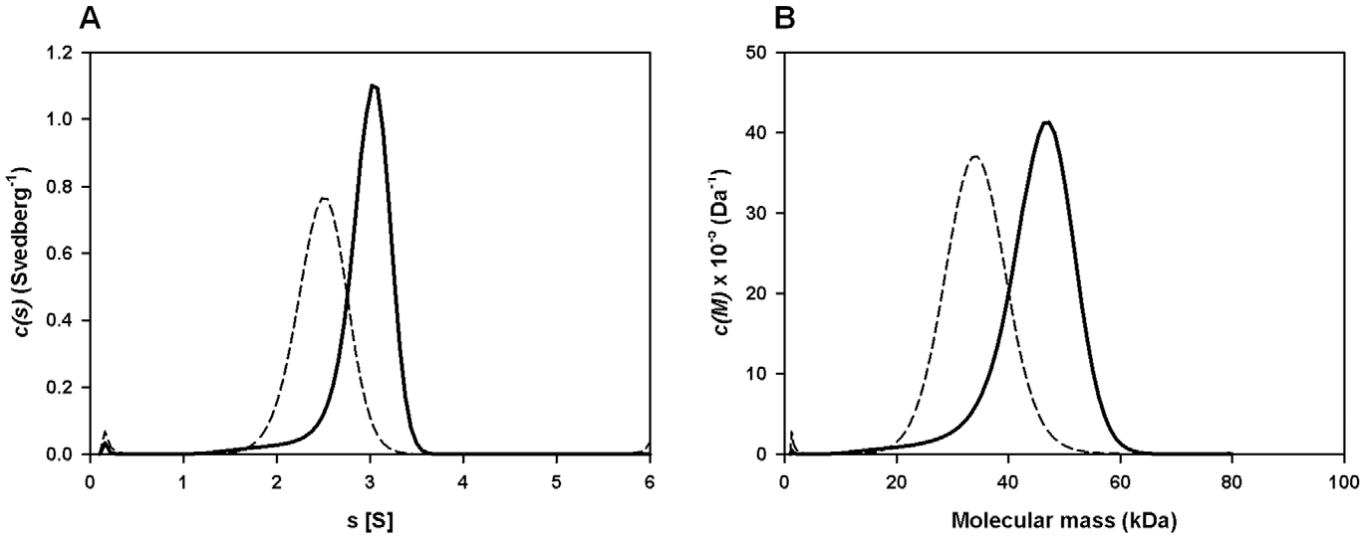
Sedimentation velocity analysis of recombinant FhHDM-1. (A) Continuous size-distribution analysis, *c(s)*, plotted as a function of sedimentation coefficient for recombinant FhHDM-1 at pH 4.5 (solid line) and pH 7.3 (dashed line). Continuous size-distribution analysis was performed using the program SEDFIT [29]–[31] employing 100 sedimentation coefficients ranging from 0.1 S to 6.0 S and at a confidence level (F-ratio)  = 0.95. (B) Continuous mass, *c(M)*, distribution plotted as a function of molecular mass (kDa) for recombinant FhHDM-1 at pH 4.5 (solid line) and pH 7.3 (dashed line). Continuous mass-distribution analysis was performed using SEDFIT with 100 masses ranging from 1.0 kDa to 80 kDa and at a confidence level (F-ratio) = 0.95.

Despite having a molecular mass of 8 kDa, native FhHDM-1 is found within a high molecular mass fraction (PI; >200 kDa) following gel filtration of adult *Fasciola* secretory proteins (Figure 1A and B). Therefore, analytical ultracentrifugation was performed to investigate the quaternary structure of recombinant FhHDM-1 (*M_r_* = 9277.6) in aqueous solution. Sedimentation velocity studies were employed at an initial protein concentration of 0.1 mg/ml at pH 4.5 and pH 7.3. The absorbance versus radial position profiles of FhHDM-1 at pH 4.5 (Figure S1A, bottom panel) and pH 7.3 (Figure S1B, bottom panel) show a predominantly single sedimenting boundary suggesting the samples are primarily homogeneous. However, there is a small proportion of a slower sedimenting boundary in the sample buffered at pH 4.5 (Figure S1A, bottom panel). To confirm the presence of multiple species, the absorbance versus radial position data at different time points were fitted to a continuous sedimentation coefficient distribution model [29]–[31] (Figure 5A). This yielded excellent fits for all samples as represented by the random distribution of residuals (Figure S1A and B, top) and the excellent statistical parameters resulting (i.e. rmsd values <0.0063 and Runs test Z values <8.8). From the ordinate maximum of the distributions shown in Figure 5A, the standardized sedimentation coefficient (*s*_20,w_) of FhHDM-1 at pH 7.3 is 2.5 S, whilst at pH 4.5, the *s*_20,w_ values of the two peaks observed in the *c(s)* distribution (Figure 5A, Table S2) are 3.1 S (major species) and 2.0 S (minor species). The equivalent continuous mass [*c(M)*]-distribution analyses of the same data (Figure 5B) suggest that the 2.5 S species at pH 7.3 is a trimer, whereas the 2.0 S species and predominant 3.1 S species at pH 4.5 correspond to dimers and pentamers, respectively (Figure 5B, solid line; Table S2). However, the broad *c(s)* and *c(M)* distributions at pH 4.5 is more likely to indicate that the sample exists as a mixture of dimers, trimers, tetramers, pentamers and potentially hexamers (Figures 5A and 5B, solid lines). In contrast, recombinant FhHDM-1 potentially exists as dimers, trimers and/or tetramers in solution at pH 7.3 (Figures 5A and 5B, dashed lines). Although the exact oligomeric identities of the species are beyond the resolution of the sedimentation velocity experiment, the data summarized in Figure 5 nevertheless demonstrates that FhHDM-1 has a greater propensity to form higher order oligomers at acidic pH compared to neutral pH.

### FhHDM-1 exhibits structural similarity to human defence molecules

The unusual helical nature of FhHDM-1 provided the impetus to conduct a bioinformatic search for small molecules with a similar structure. Helical wheel analysis (http://kael.net/helical.htm) revealed the presence of an amphipathic helix within this C-terminal region for all the trematode HDMs; for FhHDM-1 this is comprised of residues 69-89 (Figure 2A and B). This is supported by the CD spectroscopy studies (Figure 4B). This amphipathic helix is found in many small peptide antimicrobials and defensins [32]. Further probing discovered a striking structural similarity between the HDMs and the human defence molecule CAP18. CAP18 is larger than FhHDM-1 (170 as opposed to 90 residues) but regions of conservation exist between the two molecules throughout their lengths. Most importantly, the C-terminal regions of both molecules exhibit high conservation, with a signature motif EKI[X_9_]R[X_2_]D[X]L. It is known that CAP18 is secreted and undergoes cleavage in this C-terminal region by endogenous proteases to release the bioactive 37-residue peptide LL-37 containing an amphipathic helix. As shown in Figure 2, this helical structure is also found in FhHDM-1 and conserved in other trematode HDMs.

### *F. hepatica* cathepsin L1 cysteine protease releases bioactive FhHDM-1 peptides

**Figure 6:**
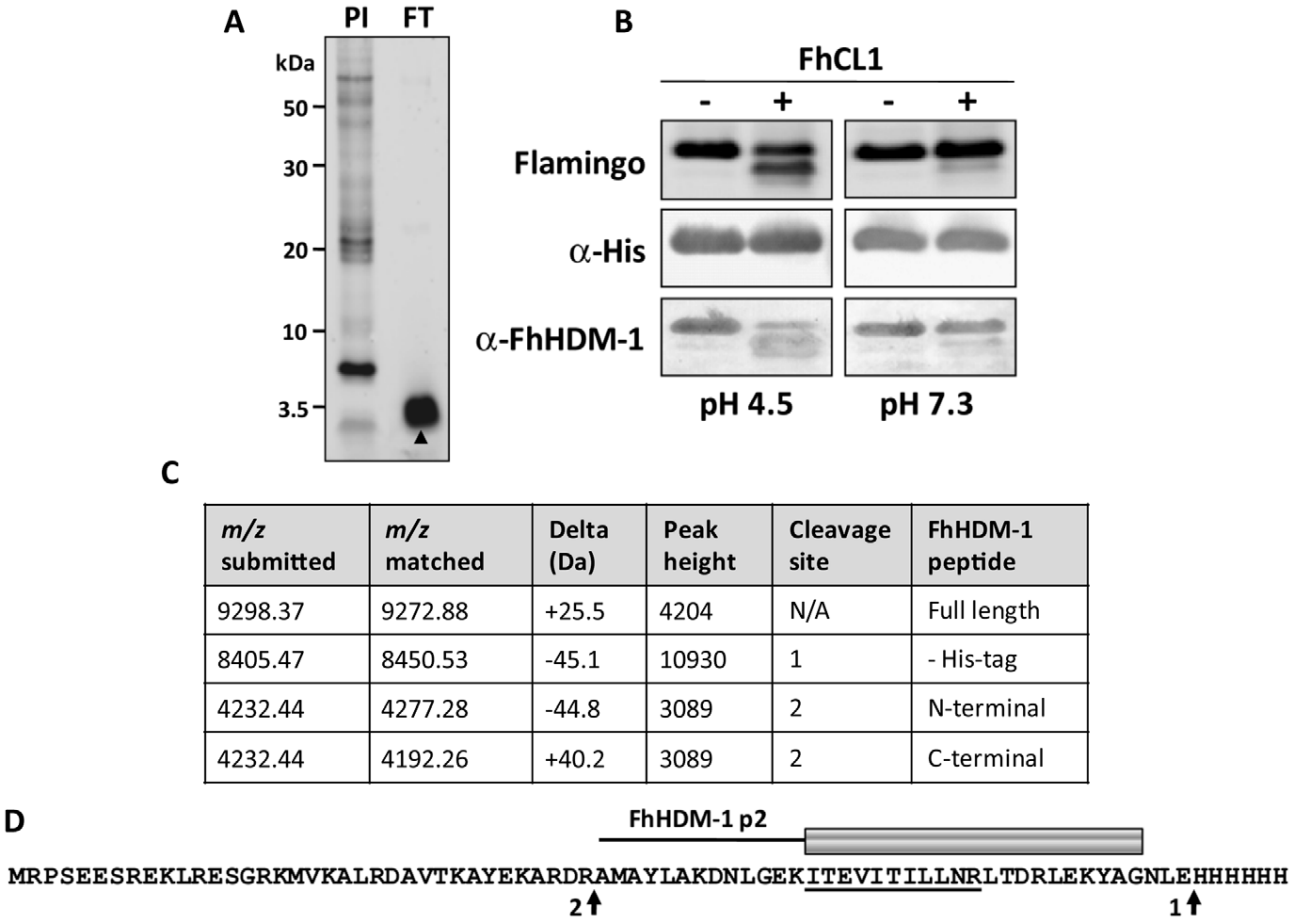
FhCL1 processes FhHDM-1 at low pH. (A) Total secretory proteins from adult *F. hepatica* were concentrated from culture supernatants using a 3 kDa cut-off filter. 10 µg of the flow-through (FT) was analysed on a 4–12% Bi-Tris gel and stained with Flamingo fluorescent protein stain. The FT comprised a single prominent band (∼ 3.5 kDa) that was identified by LC-MS/MS as an FhHDM-1 fragment (high-scoring peptide ITEVITILLNR; *m/z* 642.93, underlined in D). N-terminal sequencing of this band was unsuccessful. (B) To investigate whether *F. hepatica* cathepsin L1 (FhCL1) can process FhHDM-1, 50 µg recombinant FhHDM-1 was incubated with 1 µg recombinant FhCL1 [35] in either 0.1 M sodium acetate (pH 4.5) or 0.1 M sodium phosphate (pH 7.3) each containing 1 mM EDTA and 1 mM DTT. Reactions were performed ± FhCL1 for 3 h at 37°C and stopped by the addition of E-64 (10 µM). Samples were analysed on 4–12% Bis-Tris gels and blots were probed with anti-His or anti-FhHDM-1 antibodies. (C) The pH 4.5 reaction in the presence of FhCL1 shown in (B) was analysed by MALDI-TOF MS. The major masses detected correspond to the full length recombinant FhHDM-1 ± the C-terminal His-tag (*m/z* 9272.88 and 8450.53 respectively) and two fragments (both *m/z* 4232.44) created by a single cleavage after Arg^56^ (native peptide numbering). (D) The putative FhHDM-1 cleavage sites are arrowed. Based on this, the synthetic peptide FhHDM-1 p2 was designed (shown as a cartoon above the primary sequence of recombinant FhHDM-1). Whilst trypsinising recombinant FhHDM-1 considerably reduced its interaction with LPS, boiling had no effect.

To examine whether FhHDM-1 is processed to release peptides similar to LL-37, *F. hepatica* secretory proteins collected from *in vitro* culture supernatants were concentrated using 3 kDa molecular weight cut-off filters. The flow-though from these filters contained a single major band of ∼3.5 kDa as shown by reducing 4–12% Bis-Tris gel electrophoresis (Figure 6A). Although attempts to obtain N-terminal sequence information from this band were unsuccessful, a high-scoring FhHDM-1 peptide (ITEVITILLNR; *m/z* 642.93) was identified by LC-MS/MS analysis of trypsin digests of the 3.5 kDa band (Figure 6D). These data indicate that, like human CAP18, native FhHDM-1 is processed into smaller peptide fragments by parasite proteases. The most likely candidate for this processing event are the cathepsin L proteases, which we have previously shown are major components of the secretory products of *F. hepatica* and other trematodes [33], [34].

To investigate whether *F. hepatica* cathepsin L1 (FhCL1) can process FhHDM-1, recombinant FhHDM-1 was incubated with recombinant FhCL1 [35] at pH 4.5 or pH 7.3. Analysis of the reactions by SDS-PAGE showed that, at pH 4.5, the 9.2 kDa band corresponding to full-length recombinant FhHDM-1 had decreased in intensity concurrent with the appearance of a number of smaller protein fragments, notably, a prominent band with a molecular mass of ∼8.5 kDa (Figure 6B). The putative degradation products did not react with an anti-His antibody suggesting that the C-terminal region of the recombinant FhHDM-1 had been removed. Furthermore, an anti-FhHDM-1 antibody detected a number of the smaller protein fragments, suggesting that the full-length protein had been cleaved by FhCL1 at pH 4.5. In contrast, FhHDM-1 was not processed by FhCL1 under identical incubation conditions at pH 7.3 (Figure 6B).

The FhHDM-1 fragments produced by cathepsin L were analysed by MALDI TOF MS. Several minor peaks were found, but the major masses detected were matched to full-length recombinant FhHDM-1 ± the C-terminal His-tag (m/z 9272.88 and 8450.53, respectively) and a 37-amino acid C-terminal fragment (m/z 4232.44) created by cleavage after Arg_56_ (native peptide numbering; Figure 6C). Whilst the N-terminal fragment created by cleavage after Arg_56_ also matched this mass (Figure 6C), the presence of a C-terminal FhHDM-1 peptide in the ∼3.5 kDa band found in the Vivaspin filter flow-through supports its identification as the C-terminal fragment. Based on this putative cleavage site, a 34-residue synthetic peptide (FhHDM-1 p2) corresponding to this C-terminal region of the molecule was designed for subsequent analysis (Figure 6D). It is noteworthy that the cathepsin L cleavage site that produces the FhHDM-1 p2 peptide is, based on primary sequence alignments, only one amino acid N-terminal to the proteolytic cleavage of human CAP18 that releases the bio-active LL-37 peptide (see Figure 2).

### Recombinant FhHDM-1 and FhHDM-1 p2 bind LPS

**Figure 7:**
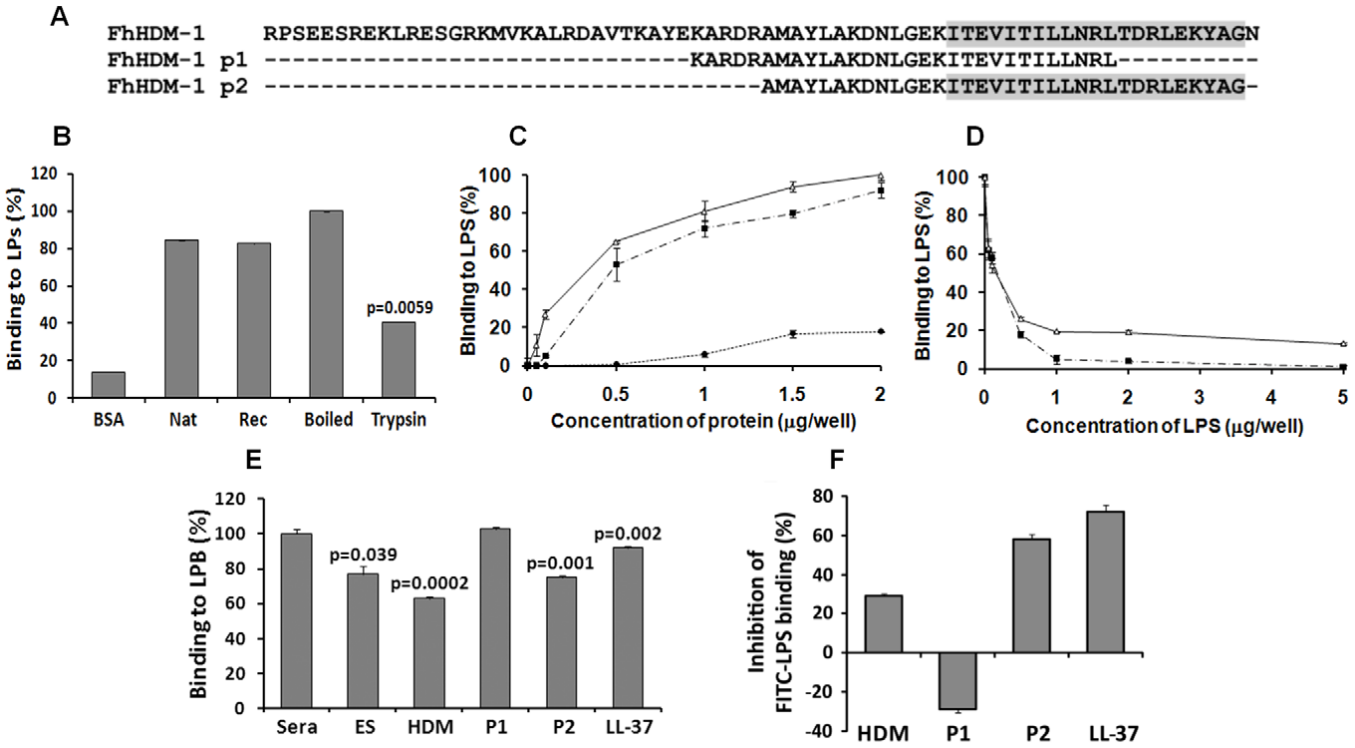
LPS neutralisation by FhHDM-1. (A) Alignment of full-length FhHDM-1 with peptide 1 (FhHDM-1 p1) and peptide 2 (FhHDM-1 p2). The conserved C-terminal amphipathic helix is shaded in grey. (B) The ability of native and recombinant FhHDM-1 to bind LPS was investigated by incubating the proteins (2 µg/well) in an LPS-coated (100 ng/well) microtitre plate. Bound proteins were detected by ELISA using rabbit anti-FhHDM-1 as a primary antibody. BSA was used as a baseline control. Whilst trypsinising the recombinant FhHDM-1 significantly reduced the LPS interaction, boiling had no effect. (C) The ability of recombinant FhHDM-1 (Δ), FhHDM-1 p1 (•) or FhHDM-1 p2 (▪) to bind to LPS was investigated by incubating a range of concentrations of proteins (0.02–2 µg/well) in this assay. (D) FhHDM-1 or derived peptides (0.1 µg) were incubated in the presence of LPS (0.05-5 µg/well) and bound peptides measured as described above. Binding of peptides to the LPS-immobilised plates was expressed as a percentage of that measured for 2 µg (for panel C) or 0.1 µg (for panel D) of FhHDM-1. Data are the means ± SD from three separate experiments. (E) FhHDM-1 and FhHDM-1 p2 but not FhHDM-1 p1 reduced the interaction between LPS and LBP as effectively as LL-37. LPS-coated microtitre plates were incubated with 5 µg/well of *F. hepatica* ES, LL-37, FhHDM-1 or derived peptides for 1 h prior to the addition of 10% human sera in PBS. Interaction of LBP with LPS was measured by ELISA using an anti-LBP primary antibody and expressed as a percentage of that detected for 10% sera in the absence of added peptides. Data are the mean ± SD of three separate experiments. Statistical significance was calculated using the student t-test and represent a comparison to the binding of 10% sera to immobilised LPS. (F) Binding of FITC-conjugated LPS to RAW264.7 cells was inhibited by LL-37, FhHDM-1 and peptides. RAW264.7 cells (5×10^5^cells/ml) were incubated with 100 ng/ml of FITC-conjugated LPS in the presence of FhHDM-1, FhHDM-1 p1, FhHDM-1 p2 and LL-37 (5 µg/ml) in RPMI 1640 containing 10% FBS for 20 min at 4°C. The binding of FITC-LPS was analysed by flow cytometry. Values represent percentage inhibition of FITC-LPS binding compared to cells in the absence of peptides. Data are the mean fluorescence ± SD of three independent experiments.

A major function of human LL-37 is the neutralisation of bacterial LPS. Given the structural similarity between FhHDM-1 and CAP18/LL-37, the ability of FhHDM-1 and derived peptides to likewise bind LPS from *E. coli* 0111:B4 was determined (Figure 7B and C). Both full-length native and recombinant FhHDM-1 bound to LPS via specific interaction with its secondary structure. The specificity of this interaction was demonstrated by an un-related recombinant protein (expressed/purified in the same manner as FhHDM-1) that displayed less than 25% of the LPS binding shown by FhHDM-1 (data not shown). Furthermore, recombinant FhHDM-1 and the C-terminal peptide FhHDM-1 p2 (containing the complete amphipathic helix) bound to LPS in a concentration-dependent manner. However, peptide FhHDM-1 p1, in which the amphipathic helix is truncated (Figure 7A), did not bind to LPS. The specific interaction between FhHDM-1 and LPS was supported by experiments in which FhHDM-1 and FhHDM-1 p2 were mixed with increasing concentrations of LPS during the ELISA. As shown in Figure 7D, both molecules bound to free LPS in solution and were therefore unable to bind LPS immobilised on the ELISA plate.

### Recombinant FhHDM-1 and FhHDM-1 p2 block the interaction of LPS with LPS-binding protein (LPB)

The ability of FhHDM-1 and derived peptides to block the interaction of LPS with LPB was assessed by ELISA using an anti-LBP primary antibody. Since it is a well-characterised α-helical defence peptide, human LL-37 was used for comparison. As shown in Figure 7E, *F* *. hepatica* ES (p = 0.039), full-length FhHDM-1 (p = 0.0002) and FhHDM-1 p2 (p = 0.001) significantly reduced the interaction between LPS and LBP as effectively as LL-37 (p = 0.002). However, FhHDM-1 p1 did not block the interaction of LPS with LPB. Thus, the ability of FhHDM-1 to block the interaction of LPS with LPB is mediated by its conserved C-terminal domain.

### Recombinant FhHDM-1 and FhHDM-1 p2 inhibit binding of FITC-LPS to RAW264.7 macrophages

The effect of FhHDM-1 and derived peptides on the binding of FITC-LPS to CD14+ cells was determined by flow cytometry of the murine macrophage cell line RAW264.7. The assay was performed at 4°C to inhibit endocytosis, thus ensuring that only cell-surface interactions were observed. FITC-LPS bound to RAW264.7 cells strongly in the absence of parasite molecules or LL-37 and this binding was inhibited (70% inhibition) by LL-37 (Figure 7F). FhHDM-1 p2 (58% inhibition) and full-length FhHDM-1 (29% inhibition) also considerably reduced FITC-LPS binding when used at 5 µg/ml (Figure 7F). In contrast, incubation with FhHDM-1 p1 increased FITC-LPS binding to the cell surface by 29%.

### Recombinant FhHDM-1 and FhHDM-1 p2 prevent LPS-induced inflammatory response in mice

**Figure 8:**
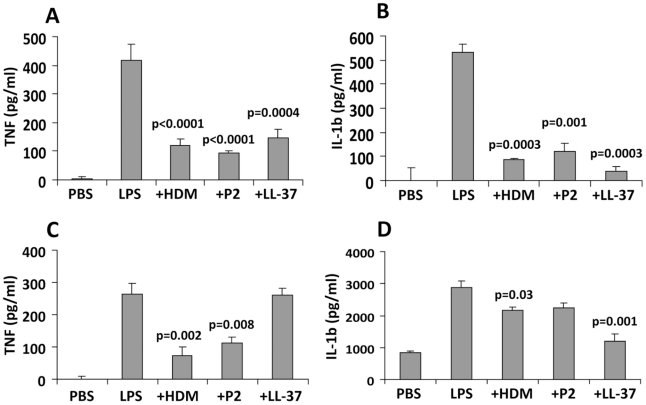
FhHDM-1 protects mice from LPS-induced inflammation. (A) BALB/c mice were injected intra-peritoneally with 1 µg of LPS alone or combined with 1 µg of FhHDM-1, FhHDM-1 p2 or LL-37. Two hours later, sera was collected and serum levels of TNF and (B) IL-1β measured by ELISA. (C) Peritoneal macrophages were isolated, cultured unstimulated in media overnight and then levels of TNF and (D) IL-1β in the culture measured by ELISA. Data are the mean ± SD of six mice in each group. Statistical significance represents a comparison to the levels of cytokines secreted by mice given LPS only.

By inhibiting the interaction between bacterial LPS and macrophages, FhHDM-1 and derived peptides are predicted to prevent the activation of an inflammatory immune response. To investigate this, BALB/c mice were injected intra-peritoneally with 1 µg of LPS alone or LPS combined with 1 µg of FhHDM-1, FhHDM-1 p3 or LL-37. Serum was collected 2 h later, and levels of the pro-inflammatory mediators TNF and IL-1β were measured by ELISA. As expected, a single dose of LPS increased the levels of both TNF and IL-1β in circulation (Figure 8A and 8B). However, when mixed with FhHDM-1, FhHDM-1 p2 or LL-37, LPS failed to induce an equivalent immune response, with levels of serum TNF and IL-1β significantly reduced (Figure 8A and 8B). Because macrophages are the main source of pro-inflammatory mediators in this murine model of inflammation, we examined whether the decrease in circulating cytokines could be attributed to a reduction in the activation of macrophages. Peritoneal macrophages were isolated from treated mice (LPS ± FhHDM-1, peptides or LL-37 as described above), and the quantity of TNF and IL-1β secreted into culture media over 16 h was measured by ELISA. Correlating with the sera data, macrophages isolated from mice injected with LPS alone showed elevated levels of both TNF and IL-1β compared to PBS-treated mice (Figure 8C and 8D) whereas production of TNF was decreased in the presence of FhHDM-1 or FhHDM-1 p2. Interestingly, LL-37 did not have a significant effect on LPS-induced secretion of TNF from macrophages in this assay (Figure 8C). This contrasts with previous studies [36]–[38] but may be due to a number of experimental differences such as the doses of LPS and LL-37 given, the serotype of LPS, and the time of cellular isolation. In addition, the full-length recombinant FhHDM-1 or LL-37 (but not FhHDM-1 p2) also reduced the release of IL-1β from peritoneal macrophages in response to LPS (Figure 8D).

## Discussion

Using proteomics and database mining we have identified a novel family of secretory proteins from trematode pathogens (termed helminth defence molecules; HDMs) that exhibit similar biochemical and functional characteristics to human defence peptides.

Host defence (also termed antimicrobial) peptides represent an evolutionarily conserved component of innate immunity [39]. Their potent antimicrobial activity has led to suggestions that they are involved not only in suppressing inflammation in the presence of pathogenic challenge, but also in maintaining homeostasis by limiting inflammatory responses that could otherwise be triggered by the presence of commensals [40]. They are widely distributed in nature, from insects and plants to highly evolved animal species with more complex immune systems [41]. Although the peptides identified to date show considerable diversity in their lengths, structures and activities, they are all generally amphipathic, possessing both cationic (positively charged) and hydrophobic faces [42], [43]. These features bestow the peptides with antimicrobial activity by facilitating interactions with negatively charged microbial cell membrane components (e.g., phospholipids), thereby increasing membrane permeability and resulting in microbial death [44]. Owing to the lack of primary sequence similarity [32], [45], peptides are broadly classified based on secondary structure as cathelicidins (linear α-helical peptides), defensins (β-strand peptides connected by disulfide bonds), and bactenecins (loop peptides) [46]. Using this classification, the 8 kDa protein (FhHDM-1) secreted by *F. hepatica* could be termed a cathelicidin as CD spectroscopy studies indicate that both native and recombinant FhHDM-1 have a high propensity to adopt α-helical structure in the presence or absence of helix-stabilising agents in both neutral and acidic pH conditions.

In humans, the most widely studied cathelicidin LL-37 is expressed by neutrophils, macrophages and mucosal epithelial cells in response to stimulation by microbes and proinflammatory mediators [45], [47]. LL-37 is secreted as an inactive precursor protein known as CAP18 that undergoes cleavage by endogenous proteases to release the bioactive 37-residue peptide LL-37 [48], [49]. Similarly, the secreted FhHDM-1 can be processed by the major cysteine protease (cathepsin L1; FhCL1) from *F. hepatica* to release a C-terminal peptide fragment. Despite having activity over a broad pH range [50] FhCL1 could only process FhHDM-1 at pH 4.5, which is similar to the pH of the *Fasciola* gut lumen. Similarly, FhCL1 can only digest host haemoglobin at pH≤4.5 as relaxation of the haemoglobin structure at acidic pH makes it susceptible to proteolysis [50]. It is unlikely that local relaxation of the FhHDM-1 structure accounts for its proteolysis at low pH since it adopts almost identical solution structures at both pH 4.5 and pH 7.3. Moreover, full-length FhHDM-1 and its C-terminal fragment were both found in native fluke secretions, suggesting that not all FhHDM-1 molecules are cleaved in the parasite gut. The greater propensity of FhHDM-1 to oligomerise at low pH (predominantly existing as pentamers) may render individual FhHDM-1 molecules inaccessible to the active site of FhCL1 thus offering some degree of protection against proteolysis. Whilst FhCL1 is the most abundant protease secreted by adult flukes, is it possible that other cathepsin cysteine proteases (e.g. FhCL2, FhCL5 and cathepsin B) could also process FhHDM-1 *in vivo.* It is also possible that FhHDM-1 may be cleaved by host proteases at neutral pH. This would be an ideal mechanism for regulating the activity of the secreted parasite molecule until it reaches its site of action on/within host cells.

Residues 13–34 of LL-37 form an amphipathic helix that anchors the peptide to phospholipid membranes via interaction with its hydrophobic face [48], [51] and is important for its antimicrobial ability [3]. In addition to these potent bactericidal activities, LL-37 can also bind to LPS and neutralize its biological activities. Among the host defence peptides, only the cathelicidin CAP18-derived peptides (human and rabbit) and CAP11 display an ability to bind to LPS [36], [52], [53]. In contrast, defensins exhibit little, if any, LPS-binding activity [36], [54]. It has been suggested that it is the amphipathic helical structure of the cathelicidin peptides that is critical for interacting with LPS [55], [56]. Indeed, LL-37-derived peptides covering residues 13–34 are as effective as full-length LL-37 with respect to LPS binding [57]. The 34-residue C-terminal peptide of FhHDM-1 released by cleavage with FhCL1 contains a 21-residue amphipathic helix which confers a striking structural parallel with the bioactive LL-37 peptide. In addition, a peptide (FhHDM-1 p2) derived from the C-terminal of FhHDM-1 containing the complete amphipathic helix binds *E. coli* LPS in a concentration-dependent manner. In stark contrast, a second peptide, FhHDM-1 p1, in which the amphipathic helix is truncated, did not bind LPS. Although recognising FhHDM-1, sera isolated from sheep at 4 weeks and 8 weeks post-infection with *F. hepatica* did not block binding of FhHDM-1 to LPS (data not shown). This is likely as a result of the sera binding the non-amphipathic region of the molecule, and the amphipathic helix remaining free to interact with LPS. Thus, the amphipathic helix of FhHDM-1, like LL-37, is a key functional determinant necessary for its biological properties.

Bacterial LPS is recognized as a key molecule in the pathogenesis of endotoxin shock associated with gram-negative bacterial infections [2], [58], [59]. By binding to LPS, LL-37 successfully neutralises its biological activities [52]. Similarly, FhHDM-1 targets key stages in LPS-mediated cell signalling. The first event in the recognition of microbial infection is the transfer of LPS to cellular CD14 by serum LPS-binding protein (LBP). This LPS-CD14 complex then initiates downstream signalling via interaction with cellular TLRs [60], which results in the secretion of inflammatory mediators. By binding directly to LPS, FhHDM-1 and FhHDM-1 p2 blocked the interaction of LPS with LBP, thus effectively reducing the number of LPS molecules that are targeted to the TLR signalling complex on the macrophage cell surface. This in turn prevents LPS-induced activation of macrophages. Therefore, FhHDM-1, and its conserved C-terminal region, just like human LL-37, impair LPS signalling and protect against harmful immune responses by reducing the release of inflammatory mediators from macrophages. In contrast, FhHDM-1 p1 increased FITC-LPS binding to the surface of RAW264.7 macrophages by 29%. Enhanced LPS binding to immune cells has previously been observed for other proteins such as bactericidal/permeability increasing protein [61] and surfactant protein A [62] but the molecular mechanism of this phenomenon for any of these immunomodulatory peptides/proteins has yet to be determined. One possible explanation is that some molecules induce mobilization (and surface expression) of LPS-binding proteins from intracellular vesicles [63].

By preventing the activation of innate immune responses by LPS, the helminth parasite enhances the survival of the host and thus its own longevity. As mentioned in the introduction, the translocation of intestinal bacteria into circulation is common during many helminth infections [11], [13]–[15]. Despite this movement of enteric microbes, fatal septicaemia during infection with trematodes including *S. mansoni* is not a common occurrence [64]. Indeed, there are, as yet, no reports of such in intestinal nematodes. In addition, in areas endemic for helminth parasites, co-infection with gram-negative bacteria, most commonly *Salmonella* sp., is common [65]. This results in a protracted clinical course of bacterial disease and yet patients show atypical symptoms of typhoid fever displaying only intermittent fever and chills [66], [67]. The reason why the helminth-infected host does not respond to such increases in potentially harmful microbes is not fully understood. One explanation given is that the conversion of macrophages to an alternatively activated phenotype during helminth infection renders them unresponsive to subsequent Th1 activation [68]. However, other reports suggest that macrophages are not terminally differentiated but display some degree of plasticity [69]. Moreover, despite long-term exposure to Th2 cytokines in vivo during a helminth infection, macrophages are nevertheless responsive to LPS, developing a classically activated phenotype characterised by the expression of iNOS [70]. Other studies demonstrate that IL-4 and IL-13 can prime macrophages for secondary microbial challenge leading to enhanced secretion of proinflammatory cytokines [71]. Therefore, during a helminth infection it is likely that a wide spectrum of macrophage phenotypes exists, displaying varying degrees of responsiveness to bacterial ligands. We propose that the active secretion of FhHDM-1 by the parasite throughout its time in the mammalian host ensures that potentially lethal LPS, either from intestinal flora or from microbial co-infections, is neutralised and that LPS-mediated activation of macrophages is controlled. Consequently, excessive inflammatory responses are avoided and the survival of the host, and therefore the parasite, are prolonged.

It is noteworthy that the HDMs show homology with the C-terminal region of the Sm16 protein from *S. mansoni* and its *S. japonicum* homologues [72], [73]. Like FhHDM-1, Sm16 forms oligomers, modulates LPS-mediated TLR signalling in human monocytes (albeit via a different mechanism to the HDMs) and its biological activity is mediated by its C-terminal region [72]. Thus, it is possible that the HDMs and the Sm16-like molecules evolved from a common ancestral protein and represent sub-clades of a larger family of helminth innate cell modulators. Our discovery of a family of HDMs that is conserved amongst medically-important trematode pathogens that modulate the immune response via molecular mimicry of host defence peptides provides a common mechanism for the anti-inflammatory properties of helminth infection.

While many cationic amphiphilic compounds are being developed as therapeutics based on their potent antimicrobial activity [74], [75], only derivatives of LL-37 have been proposed as attractive candidates for anti-sepsis therapies [32]. Our discovery of a novel family of HDMs derived from worm parasites provides a new pool of bio-active peptides that may have unique pharmacological properties with prospects for the development of novel anti-inflammatory therapeutics.

## Materials and Methods

### Preparation of adult *F. hepatica* secretory proteins

Mature *Fasciola hepatica*, were recovered from the liver tissue and bile ducts of Merino sheep 16 weeks after an experimental infection with 200 infective stage larvae (metacercariae) and washed in pre-warmed (37°C) PBS pH 7.3. Flukes were then transferred to pre-warmed (37°C) RPMI 1640 medium (Invitrogen) containing 2 mM L-glutamine, 30 mM HEPES, 0.1% (w/v) glucose and 2.5 µg/ml gentamycin and incubated for 8 h at 37°C. The culture medium containing *F. hepatica* secretory proteins was pooled and concentrated using Vivapsin columns (VivaScience) with a 3 kDa molecular weight cut-off to a final concentration of 1 mg/ml and stored in aliquots at −20°C. The flow-through from the Vivaspin columns was concentrated to approximately 1 mg/ml using a Concentrator 5301 (Eppendorf) and stored in aliquots at −20°C until use.

### Biochemical purification of native FhHDM-1 from *F. hepatica* secretions

Concentrated *F. hepatica* secretory proteins (1 mg) were separated by size exclusion chromatography (gel filtration) using a Superdex 75 HiLoad 16/60 column (GE Healthcare) attached to a BioLogic LP system (BioRad). Samples were resolved with PBS, pH 7.3, at a flow rate of 1 ml/min. The eluant was monitored at 220 nm and 0.5 ml fractions were collected over time. The major peak with a calculated molecular mass >200 kDa (termed PI; [18]) that was observed following gel filtration was further resolved by reverse phase high performance liquid chromatography (RP-HPLC) using an Agilent 1200 HPLC (Agilent Technologies, Palo Alto, CA) fitted with a Phenomenex Jupiter 5µ C_4_ 300 Å column (150×2.0 mm). The column flow rate was 100 µL/min and samples were eluted using a gradient of buffer A (0.1% TFA and 2% acetonitrile) and buffer B (0.1% TFA and 98% acetonitrile). The eluted proteins were detected at 220 nm and 0.5 ml fractions were collected by an automated fraction collector based on peak detection.

### Synthetic FhHDM-1 peptides

FhHDM-1 peptide 1 (FhHDM-1 p1) is 30 residues in length, corresponding to residues 51–80 (KARDRAMAYLAKDNLGEKITEVITILLNRL), and was designed as a truncation of the C-terminal amphipathic helix. FhHDM-1 p2 is 34 residues in length and corresponds to residues 56–89 (AMAYLAKDNLGEKITEVITILLNRLTDRLEKYAG). It was designed to match the C-terminal fragment of FhHDM-1 containing the complete amphipathic helix that is released following cleavage by FhCL1. LL-37 and peptides derived from FhHDM-1 were synthesised by GL Biochem (Shanghai, China).

### N-terminal sequencing and LC-MS/MS

Protein samples from RP-HLPC fractions were analysed using NuPage® Novex® 4–12% Bis-Tris gels (Invitrogen). NuPage® LDS sample buffer plus Sample Reducing Agent (Invitrogen) were added to the samples and heated at 95°C for 5 min prior to electrophoresis. Proteins were then transferred to polyvinylidene fluoride (PVDF) immobilon-P membranes (Millipore) at 120 mA for 45 min. The membranes were washed with distilled water and stained with 0.025% Coomassie Brilliant Blue R-250 in 40% methanol, 10% acetic acid. Selected protein bands were subjected to 5 cycles of N-terminal (Edman) sequencing using an Applied Biosystems 494 Procise Protein Sequencing System at the Australian Proteome Analysis Facility (Sydney, Australia).

Liquid chromatography tandem mass spectrometry (LC-MS/MS) analysis was performed as described previously [34], [76]. Briefly, individual gel bands were cut into smaller pieces (approximately 1 mm^2^) and reduced and alkylated with 5 mM tributylphosphine and 20 mM acrylamide (Sigma) in 100 mM NH_4_HCO_3_ for 90 min. The excised sections were digested in-gel with trypsin (Sigma Proteomics grade) and the peptides solubilised with 2% formic acid (Sigma) prior to analysis by LC-MS/MS using a Tempo nanoLC system (Applied Biosystems) with a C18 column (Vydac) coupled to a QSTAR Elite QqTOF mass spectrometer running in IDA mode (Applied Biosystems). Peak list files generated by the Protein Pilot v1.0 software (Applied Biosystems) using default parameters were exported to a local PEAKS (Bioinformatics Solutions Inc.) search engine and employed as queries to search a custom-made database composed of all *F. hepatica* ESTs (14,031 reads) currently available from the Wellcome Trust Sanger Centre (ftp://ftp.sanger.ac.uk/pub/pathogens/Fasciola/hepatica/ESTs/). The enzyme specificity was set to trypsin, propionamide (acrylamide) modification of cysteines was used as a fixed parameter and oxidation of methionines was set as a variable protein modification. The mass tolerance was set at 100 ppm for precursor ions and 0.2 Da for fragment ions and only one missed cleavage was allowed. Only high-scoring (>60%) peptides were considered to be significant [76].

### *F. hepatica* RNA extraction and RT-PCR

Newly excysted juvenile (NEJ) flukes were recovered from dormant metacercariae (Baldwin Aquatics, OR, USA) using a method developed in our laboratory as described by Robinson *et al*. [76]. Immature *F. hepatica* (21 day-old) were recovered from the livers of female BALB/c mice (experimentally infected with 20 metacercariae; [76]) while adult parasites were recovered from the bile ducts of Merino sheep as described above. Total RNA was prepared from *F. hepatica* NEJs, immature flukes and adult parasites using Trizol reagent (Invitrogen) according to the manufacturer's instructions. Contaminating genomic DNA was removed by treatment with DNAse I (Promega). Reverse transcription was performed with 1 µg total RNA using an oligo(dT)_15_ primer and 200 U M-MLV (H-) reverse transcriptase (Promega) at 50°C for 1 h according to the manufacturer's instructions. Aliquots of the resulting cDNA from each life-cycle stage (200 ng) were subjected to PCR amplification under the following conditions: 94°C for 1 min, 58°C for 30 s and 72°C for 1 min with a final extension at 72°C for 5 min. A total of 30 cycles were performed. The following gene-specific primers were designed using an adult *F. hepatica* EST (identifier HAN3008-1e06.p1k) and used for PCR: FhHDM-1_F1 5′-CATATGAGACCTAGCGAGGAAAGCCG-3′ and FhHDM-1_R1 5′-CTCGAGATTTCCCGCGTATTTCTCCAAG-3′. RT-PCR amplification of constitutively expressed *F. hepatica* β-actin was performed as a positive control. PCR products were separated by 1% agarose gel electrophoresis and stained with ethidium bromide.

### Expression and purification of recombinant FhHDM-1 in *E. coli*

The FhHDM-1 protein coding sequence downstream of the predicted N-terminal signal peptide was amplified using the Expand High Fidelity PCR system (Roche) with the primers FhHDM-1_F1 and FhHDM-1_R1 and cloned into the pCR2.1 TOPO vector (Invitrogen). Following DNA sequencing at the Australian Genome Research Facility (Brisbane, Australia), the insert was sub-cloned into *Nde*I/*Xho*I restriction sites of the pET21b expression vector (Novagen) and *Escherichia coli* strain BL21 transformed with the resulting constructs. Bacterial cultures (1 L) were grown in LB media supplemented with ampicillin (100 µg/ml) at 37°C until OD_600_ 0.4–0.6 and expression of the recombinant FhHDM-1 was induced by the addition of 1 mM IPTG for 4 h at 37°C. Cell pellets were collected by centrifugation at 4000 x g for 20 min at 4°C and then resuspended in lysis buffer (300 mM KCl; 50 mM KH_2_PO_4_; 5 mM imidazole). Cells were lysed by sonication at maximum power for six cycles of 10 s on/50 s off. Cleared lysate was obtained by centrifugation at 10000 x g for 30 min at 4°C. Soluble recombinant His-tagged FhHDM-1 protein was purified and desalted using the Profinia Protein Purification System (BioRad) according to manufacturer's instructions. Briefly, the cleared lysate was loaded onto a 1 mL IMAC column and washed with 6 column volumes of lysis buffer (300 mM KCl; 50 mM KH_2_PO_4_; 5 mM imidazole) followed by a second wash containing 10 mM imidazole. Protein was eluted in the same buffer containing 250 mM imidazole and loaded directly onto a 10 mL desalting column. The final desalted recombinant FhHDM-1 protein was eluted in PBS (pH 7.3) and stored in aliquots at −20°C until use. The purified full-length recombinant FhHDM-1 was also used to raise polyclonal antibodies in rabbits (Auspep, Australia).

### Processing of FhHDM-1 by FhCL1

Processing of FhHDM-1 was carried out by mixing 50 µg of the purified recombinant with 1 µg recombinant *F. hepatica* cathepsin L1 (FhCL1; [35]). The mixtures were incubated in either 0.1 M sodium acetate (pH 4.5) or 0.1 M sodium phosphate (pH 7.3) each containing 1 mM EDTA and 1 mM DTT for 3 h at 37 °C. Reactions were performed ± FhCL1 and stopped by the addition of E-64 (10 µM). Samples were analysed on 4–12% Bis-Tris gels and stained with Flamingo fluorescent protein stain (Invitrogen) or transferred to nitrocellulose membranes for immuno-detection. Blots were blocked with 5% nonfat dry milk in PBS Tween (0.05% v/v) and incubated with either a 1∶10,000 dilution of rabbit antiserum raised against recombinant FhHDM-1 or a 1∶1000 dilution of a mouse anti-His_6_ antibody (Abcam) and subsequently with alkaline phosphatase-conjugated goat anti-rabbit/mouse IgG (Sigma) for 1 h. Blots were visualised by the addition of 3,3-diaminobenzidine peroxidase substrate (Sigma).

To identify the peptide fragments resulting from FhCL1 processing of FhHDM-1, samples of the reaction performed at pH 4.5 were analysed by Matrix Assisted Laser Desorption Ionisation (MALDI) mass spectrometry at the Australian Proteome Analysis Facility. Briefly, samples were mixed 1∶1 with a-cyanohydrocinnamic acid (1 mg/mL in 90% acetonitrile containing 0.1% TFA) and spotted (1 µL) onto a MALDI plate that contained a dried matrix spot from 0.5 µL matrix. An additional spot of matrix (1 µL) was added and dried. A protein calibration spot was spotted next to the sample in the same manner. MALDI MS was performed using an AB Sciex 4800+ instrument using a Nd:YAG laser (355 nm) to irradiate the sample. Spectra were acquired in positive mode in the mass range 2,000–20,000 Da with a mass accuracy of ±50 Da. The mass spectrometer was calibrated using Bruker calibrant proteins.

### Enzyme-linked immunosorbent assay and immunoblot analysis

Serum samples were obtained from six male Merino sheep experimentally infected with *F. hepatica*. Serum samples were taken 8 weeks prior to infection with 200 metacercariae and on weeks 4, 8, 12 and 16 post-infection and used for ELISA and immunoblotting. One hundred microliters of recombinant FhHDM-1 (5 µg/ml) was dispensed into the wells of microtiter plates that were then incubated overnight at 4°C. Excess protein binding sites were blocked at 37°C for 1 h by adding 200 µl of 1% bovine serum albumin diluted in PBS containing 0.1% Tween 20 (PBST) to each well. After the wells were washed three times with PSBT, sheep sera (diluted 1∶1000) was added and the plates were incubated for 2 h at 37°C. Following another wash, 100 µl of alkaline phosphatase-conjugated anti-sheep IgG (diluted 1∶10,000) was added to each well and the plates were incubated for an additional 1 h at 37°C. After another washing step, bound antibodies were detected by the addition of 200 µl of *p*-nitrophenol phosphate (1 mg/ml) in 0.2 M Tris buffer, pH 8.0. After the colour had developed for 30 min, the plates were read on a Power Wave HT (BioTek) at 405 nm. All assays were tested in triplicate. Sera from individual animals that showed sero-positivity for FhHDM-1 by the above ELISA method were selected for immunoblot analysis. Briefly, 10 µg of recombinant FhHDM-1 or adult *F. hepatica* secretory proteins were run on reducing NuPage® Novex® 4–12% Bis-Tris gels (Invitrogen) and transferred to PVDF immobilon-P membranes (Millipore) at 120 mA for 45 min. Following transfer, the membranes were incubated in blocking solution (TSBT: 20 mM Tris–HCl, 150 mM NaCl, 1% Tween-20, pH 7.6) containing 5% skimmed milk for 3 h at room temperature. Sheep antisera were diluted 1∶1000 in TBST containing 1% skimmed milk and applied to the membranes overnight at 4°C. After washing in TSBT (4×10 min), an alkaline phosphatase-conjugated anti-sheep-IgG secondary antibody was applied to the membranes for 30 min at room temperature before detection using *p*-nitrophenol phosphate (1 mg/ml) in 0.2 M Tris buffer, pH 8.0.

### Circular dichroism spectroscopy

Circular dichroism (CD) spectra were recorded using an AVIV 410-SF CD spectrometer. Wavelength scans were performed between 190 and 250 nm in either 10 mM Tris, 50 mM NaF buffer (pH 7.3) or 10 mM sodium citrate, 50 mM NaF buffer (pH 4.5) in both the presence and absence of 30% (v/v) TFE with a sample concentration of 0.1 mg mL^−1^. Spectra were recorded in a 1 mm quartz cuvette at 20 °C. Data below 195 nm for the native FhHDM-1 sample at both pH 7.3 and pH 4.5 were removed from analyses due to low signal-to-noise. Data were analyzed using the CDPro software package [27].

### Analytical ultracentrifugation

Sedimentation velocity experiments were conducted in a Beckman model XL-I analytical ultracentrifuge at a temperature of 20°C. Samples were analysed at a concentration of 0.1 mg/ml in either 10 mM Tris, 50 mM NaF buffer (pH 7.3) or 10 mM sodium citrate, 50 mM NaF buffer (pH 4.5). Samples were loaded into a conventional double sector quartz cell and mounted in a Beckman 4-hole An-60 Ti rotor. 380 µl of sample and 400 µl of reference solution were centrifuged at a rotor speed of 40,000 rpm, and the data were collected at a single wavelength (230 nm) in continuous mode, using a step-size of 0.003 cm without averaging. Solvent density (1.0014 g/ml at pH 4.5 at 20°C and 1.0000 g/ml at pH 7.3) and viscosity (1.0077 cp at pH 4.5 and 1.0174 at pH 7.3) were determined using a Density Meter and Automated Microviscometer (Anton Paar) and estimates of the partial specific volume (0.7327 ml/g for recombinant FhHDM-1) were computed using the FhHDM-1 amino acid composition employing the program SEDNTERP [77]. Sedimentation velocity data at multiple time points were fitted to a single discrete species and a continuous size-distribution model [29]-[31] using the program SEDFIT, which is available at www.analyticalultracentrifugation.com.

### Bioinformatics and phylogenetic analysis

FhHDM-1 orthologs in other trematodes related to *F. hepatica* were identified following tBLASTn analysis of the NCBI nucleotide and EST databases (http://blast.ncbi.nlm.nih.gov/Blast.cgi) using the FhHDM-1 primary sequence as search query. Specific searches were also performed against the various *F. hepatica* (http://worm1.liv.ac.uk/blast/blast.html), *S. mansoni* (http://www.sanger.ac.uk/DataSearch/blast.shtml) and *S. japonicum* (http://function.chgc.sh.cn/sj-proteome/blastn.htm) genome and transcriptome databases. The trematode HDM primary sequences were aligned using CLUSTAL W [78]. Secondary structure prediction was performed using PSIPRED [26] and amphipathic helix prediction was performed using helical wheel analysis (http://www.kael.net/helical.htm). Phylogenetic trees were created using 17 trematode cDNA sequences that encoded full-length HDM proteins but excluding the signal peptide. The DNA sequences were initially aligned using CLUSTAL W [68] and the trees were created using the boot-strapped (1000 trials) neighbour-joining method of MEGA version 4.0 [79], using the Kimura 2 parameter model with uniform rates for all sites. The GenBank accession numbers/EST identifiers of the sequences used for alignment and phylogenetic analyses are as follows. Schistosome HDMs: FN314266, AM047184, FN314265, FN314266, FN314267, FN357430; *Fasciola*/Asian fluke HDMs: HQ456365, AF281362, AT009171, AT007125, ES416124; Sm16-like molecules: AF109180, EF127841, AY812960, AY814368, AY915075 and FN327058. The phylogenetic tree was rooted with human CAP18 (accession number NM_004345).

### Measurement of the LPS-binding activities of FhHDM-1 and derived peptides

Microtiter plates (96 well; Nunc) were coated with *E. coli* LPS (100 ng/well; serotype 111:B4; Sigma) in PBS for 3 h at 37°C, following which the plates were rinsed thoroughly under running water and air-dried overnight. After blocking excess binding sites with 1% BSA/PBS, native and recombinant FhHDM-1 or derived peptides (0.02-2.0 µg/well in PBS) were added to the plate which was then incubated for 1 h at 37°C. Binding of FhHDM-1 or peptides to LPS was detected by the addition of affinity-purified rabbit anti-FhHDM-1 (1∶5000 dilution in 0.1% BSA/PBS) for 1 h at 37°C, followed by alkaline phosphatase-conjugated goat anti-rabbit IgG (Sigma; 1∶2000 dilution in 0.1% BSA/PBS). Binding of secondary antibody was visualised by the addition of *p*-nitrophenol phosphate (Sigma; 100 µl/well) and measuring the absorbance at 405 nm. Alternatively, FhHDM-1 or derived peptides (0.1 µg/well) were added to LPS-coated plates in the presence of LPS (0.05 to 5.0 µg/well) in PBS. Bound peptide was then determined by the addition of anti-FhHDM-1 antibody as described above.

### Assay for the interaction of LPS with LBP

LPS–LBP binding was examined as described previously [80]. Briefly, PBS containing 0.1-10% mouse sera was added to an LPS-coated microtiter plate (100 ng/well) and incubated for 1 h at 37°C. Bound LBP was detected by the addition of anti-LBP antibody (Santa Cruz; 1∶500 dilution in 0.1% BSA/PBS) followed by alkaline phosphatase-conjugated rabbit anti-goat IgG (Sigma; 1∶1000 dilution on 0.1% BSA/PBS) and measuring the colour development at 405 nm after addition of *p*-nitrophenol phosphate (Sigma; 100 µl/well). To examine the effect of FhHDM-1 or derived peptides on the interaction between LPS and LBP, LPS-coated microtiter plates were pre-incubated with either *F. hepatica* ES, FhHDM-1, FhHDM-1 p1 or FhHDM-1 p2 (0.01–10 µg/ml) for 1 h at 37°C prior to the addition of 10% mouse sera in PBS and assayed as described above.

### Assay for binding of FITC-conjugated LPS to RAW 264.7 cells

RAW 264.7 cells (5×10^5^/ml) were incubated with FITC-conjugated LPS (100 ng/ml) in the absence or presence of FhHDM-1 or derived peptides (0.01 to 10 µg/ml) in RPMI 1640 containing 10% FBS for 20 min at 4°C. After cells were washed with PBS, the binding of FITC-conjugated LPS was analysed by measuring median fluorescence intensity using a LSR II flow cytometer (BD Bioscience).

### The effects of FhHDM-1 and derived peptides on endotoxin-induced inflammation

Following purification of native and recombinant FhHDM-1 by gel filtration and Ni-NTA agarose chromatography respectively, residual bacterial endotoxin was removed from the samples using RP-HPLC using the same conditions as described above. Synthetic peptides were supplied endotoxin free. Final endotoxin levels were measured using the Chromo-LAL assay kit (Associates of Cape Cod, USA) and shown to be <0.01 EU/ml. Six week-old female BALB/c mice were purchased from ARC (Perth, Australia) and maintained according to the guidelines of the University of Technology Sydney Animal Care and Ethics committee. For the analysis for endotoxin-induced inflammation, mice were intra-peritoneally injected with 1 µg of *E. coli* LPS (serotype 111:B4; Sigma) either with or without 1 µg FhHDM-1 or derived peptides. After 2 h mice were euthanized. Plasma was isolated from cardiac blood by centrifugation at 2000 x g for 10 min and levels of circulating IL-1β and TNF measured by ELISA (BD Pharmingen). Additionally, the peritoneal cavities of mice were lavaged and peritoneal macrophages isolated by adherence to plastic as previously described [81]. Macrophages were incubated overnight in RPMI 1640 supplemented with 10% FCS and supernatants were analysed for the presence of IL-1β and TNF by ELISA.

### Accession numbers

FN314266, AM047184, FN314265, FN314266, FN314267, FN357430, HQ456365, AF281362, AT009171, AT007125, ES416124, AF109180, EF127841, AY812960, AY814368, AY915075, FN327058, NM_004345.

## Supporting Information

Figure S1 Sedimentation velocity analysis of recombinant FhHDM-1. Absorbance versus radial position of recombinant FhHDM-1 at pH 4.5 (A) and pH 7.3 (B). The residuals for the resulting *c(s)* distribution best-fits are shown.
(TIF)

Table S1 Secondary structure proportions of native and recombinant FhHDM-1.
**(**DOC**)**

Table S2 Hydrodynamic properties of recombinant FhHDM-1 samples.
**(**DOC**)**
