# Supplementary figures and images for: A Family of Helminth Molecules that Modulate Innate Cell Responses via Molecular Mimicry of Host Antimicrobial Peptides
Source: PLoS Pathog. 2011 May 12;7(5):e1002042. doi: 10.1371/journal.ppat.1002042 (PMC3093369; doi:10.1371/journal.ppat.1002042)

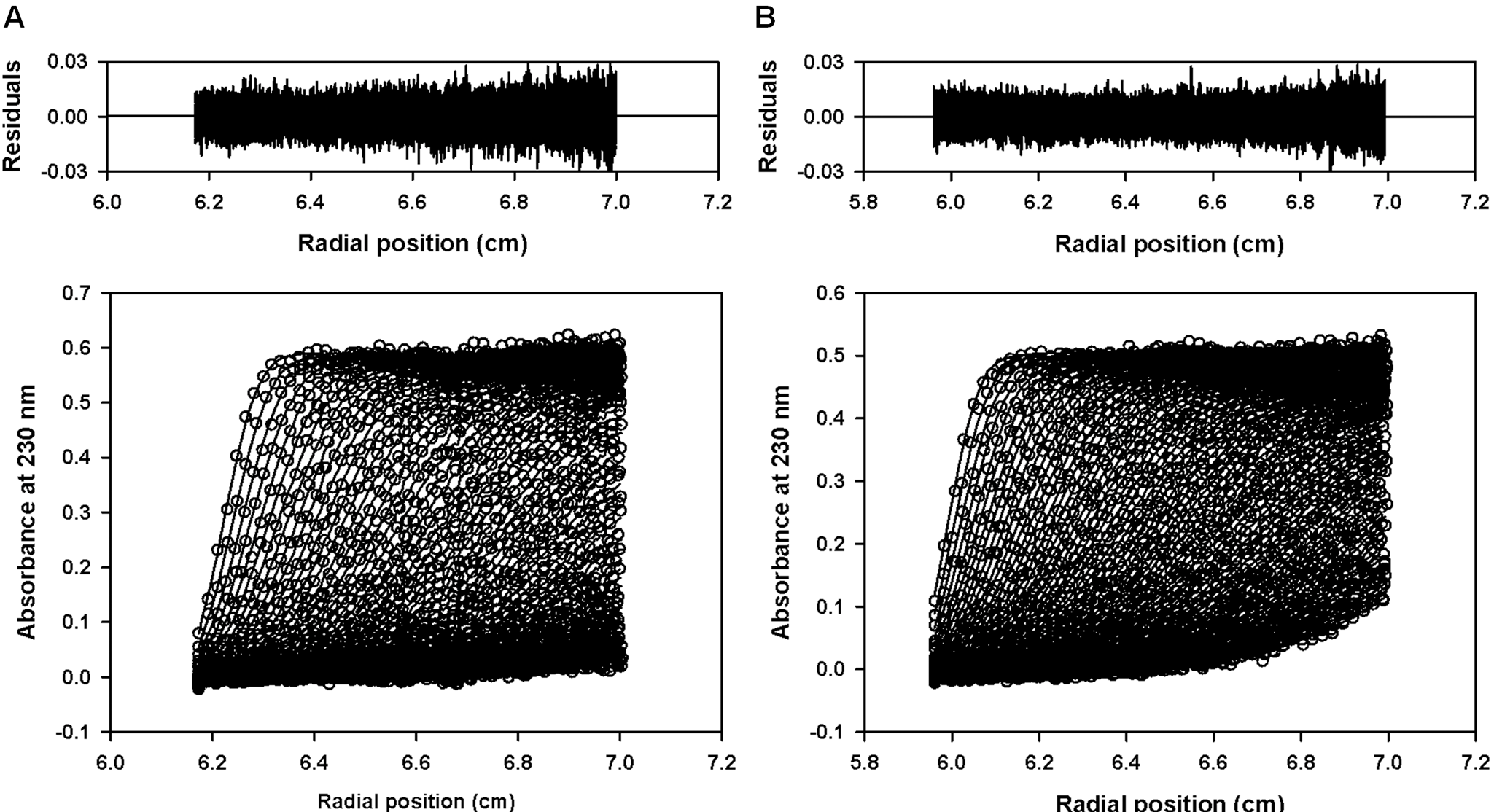

Supplement: Figure S1 — Sedimentation velocity analysis of recombinant FhHDM-1. Absorbance versus radial position of recombinant FhHDM-1 at pH 4.5 (A) and pH 7.3 (B). The residuals for the resulting c(s) distribution best-fits are shown. (TIF) [file ppat.1002042.s001.tif]
