# Supplementary material for: A Family of Helminth Molecules that Modulate Innate Cell Responses via Molecular Mimicry of Host Antimicrobial Peptides
Source: PLoS Pathog. 2011 May 12;7(5):e1002042. doi: 10.1371/journal.ppat.1002042 (PMC3093369; doi:10.1371/journal.ppat.1002042)
Supplement: Table S1 — Secondary structure proportions of native and recombinant FhHDM-1. (DOC) [file ppat.1002042.s002.doc]

**Table S1. Secondary structure proportions of native and recombinant FhHDM-1.**

| **Secondary structure** | *α_reg_*^a^ | *α_dis_*^b^ | **α_tot_^c^** | *β_reg_*^a^ | *β_dis_*^b^ | **β_tot_^c^** | **Turn^d^** | **Unordered^e^** |
| --- | --- | --- | --- | --- | --- | --- | --- | --- |
| **Recomb pH 4.5** | *45 %* | *21 %* | **66 %** | *0 %* | *2 %* | **2 %** | **7 %** | **25 %** |
| **Recomb pH 4.5 + TFE** | *57 %* | *27 %* | **84 %** | *0 %* | *0 %* | **0 %** | **1 %** | **15 %** |
| **Recomb pH 7.3** | *37 %* | *22 %* | **59 %** | *0 %* | *3 %* | **3 %** | **12 %** | **26 %** |
| **Recomb pH 7.3 + TFE** | *53 %* | *23 %* | **76 %** | *0 %* | *1 %* | **1 %** | **1 %** | **22 %** |
| **Native pH 4.5** | *53 %* | *34 %* | **87 %** | *0 %* | *6 %* | **6 %** | **7 %** | **0 %** |
| **Native pH 4.5 + TFE** | *49 %* | *30 %* | **79 %** | *1 %* | *0 %* | **1 %** | **20 %** | **0 %** |
| **Native pH 7.3** | *66 %* | *34 %* | **100 %** | *0 %* | *0 %* | **0 %** | **0 %** | **0 %** |
| **Native pH 7.3 + TFE** | *67 %* | *32 %* | **99 %** | *0 %* | *0 %* | **0 %** | **1 %** | **0 %** |

^a^ Regular α/β secondary structure

^b^ Distorted α/β secondary structure, such as residues at the ends of helices or strands

^c^ Total α/β secondary structure; the sum of regular and distorted α/β secondary structure

^d^ Residues that are in a typical turn orientation

^e^ Residues that do not fall into any of the above categories are termed “Unordered”
