# Supplementary material for: A Family of Helminth Molecules that Modulate Innate Cell Responses via Molecular Mimicry of Host Antimicrobial Peptides
Source: PLoS Pathog. 2011 May 12;7(5):e1002042. doi: 10.1371/journal.ppat.1002042 (PMC3093369; doi:10.1371/journal.ppat.1002042)
Supplement: Table S2 — Hydrodynamic properties of recombinant FhHDM-1 samples. (DOC) [file ppat.1002042.s003.doc]

**Table S2. Hydrodynamic properties of recombinant FhHDM-1 samples.**

| **Sample** | $s_{20,w}^{o}$**^1^**  **(Svedberg, S)** | ***M*^2^**  **(kDa)** |
| --- | --- | --- |
| pH 4.5 | 3.07 (major species)  1.98 (minor species) | 47.2  21.5 |
| pH 7.3 | 2.54 | 34.4 |

^1^ Standardized sedimentation coefficient taken from the ordinate maximum of the *c(s)* distribution (data not shown).

^2^Apparent molecular mass (*M*) taken from the ordinate maximum of the *c(M)* distribution (Figure S1).
